# New Tetrahydroisoquinoline Derivatives Overcome Pgp Activity in Brain-Blood Barrier and Glioblastoma Multiforme in Vitro

**DOI:** 10.3390/molecules23061401

**Published:** 2018-06-09

**Authors:** Iris Chiara Salaroglio, Elena Gazzano, Joanna Kopecka, Konstantin Chegaev, Costanzo Costamagna, Roberta Fruttero, Stefano Guglielmo, Chiara Riganti

**Affiliations:** 1 Department of Oncology, University of Torino, via Santena 5/bis, 10126, Torino Italy; irischiara.salaroglio@unito.it (I.C.S.); elena.gazzano@unito.it (E.G.); joanna.kopecka@unito.it (J.K.); costanzo.costamagna@unito.it (C.C.); 2 Department of Drug Science and Technology, University of Torino, via Pietro Giuria 9, 10125, Torino, Italy; konstantin.chegaev@unito.it (K.C.); roberta.fruttero@unito.it (R.F.)

**Keywords:** P-glycoprotein, glioblastoma multiforme, brain-blood barrier, doxorubicin

## Abstract

P-glycoprotein (Pgp) determines resistance to a broad spectrum of drugs used against glioblastoma multiforme (GB). Indeed, Pgp is highly expressed in GB stem cells and in the brain-blood barrier (BBB), the peculiar endothelium surrounding the brain. Inhibiting Pgp activity in the BBB and GB is still an open challenge. Here, we tested the efficacy of a small library of tetrahydroisoquinoline derivatives with an EC_50_ for Pgp ≤ 50 nM, in primary human BBB cells and in patient-derived GB samples, from which we isolated differentiated/adherent cells (AC, i.e., Pgp-negative/doxorubicin-sensitive cells) and stem cells (neurospheres, NS, i.e., Pgp-positive/doxorubicin-resistant cells). Three compounds used at 1 nM increased the delivery of doxorubicin, a typical substrate of Pgp, across BBB monolayer, without altering the expression and activity of other transporters. The compounds increased the drug accumulation within NS, restoring doxorubicin-induced necrosis and apoptosis, and reducing cell viability. In co-culture systems, the compounds added to the luminal face of BBB increased the delivery of doxorubicin to NS growing under BBB and rescued the drug’s cytotoxicity. Our work identified new ligands of Pgp active at low nanomolar concentrations. These compounds reduce Pgp activity in BBB and GB and improve in vitro chemotherapy efficacy in this tumor.

## 1. Introduction

Glioblastoma multiforme (GB) is considered the most common, aggressive and lethal brain tumor in the adult population, because of its highly infiltrating nature. GB usually arises from the white matter as a heterogeneous lesion, but it rapidly spreads into the surrounding brain tissue [1]. GB standard therapy is based on surgical resection, followed by radiotherapy and chemotherapy based on temozolomide. The second-line therapy is based on topoisomerase I and II inhibitors or anti-angiogenic drugs. The success of chemotherapy is limited by the tumor polyclonality, the intrinsic resistance to most chemotherapeutic drugs and the presence of blood-brain barrier (BBB) [2,3,4].

Moreover, chemotherapy is not efficient to completely eradicate tumor stem cells (SCs) that contribute to GB initiation, progression and recurrence. Indeed, SCs show a multidrug resistance (MDR) phenotype [5,6] that prevents the intracellular accumulation and efficacy of several antineoplastic drugs. The MDR phenotype of GB SCs is sustained by the high expression of adenosine triphosphate (ATP) binding cassette (ABC) transporters, such as P-glycoprotein (Pgp/ABCB1), MDR related protein 1 (MRP1/ABCC1), breast cancer resistance protein (BCRP/ABCG2) [6].

A second reason of chemotherapy failure against GB is due to the low delivery of drugs across the BBB, the microvascular endothelium that surrounds brain parenchyma. BBB is characterized by the absence of fenestrations and the presence of tight junctions (TJs) and ABC transporters [7,8]. BBB is often disrupted within GB bulk, but it is competent in the “brain-adjacent to tumor” (BAT) area, where isolated GB cells are present, and may produce local tumor recurrence or metastatic dissemination if not eradicated by chemotherapy [7]. Pgp is abundant on GB SCs and on the luminal side of BBB, and mediates the backward efflux of doxorubicin, taxanes, *Vinca* alkaloids, teniposide/etoposide, topotecan, methotrexate, imatinib, dasatinib, lapatinib, gefitinib, sorafenib, and erlotinib [8].

The presence of Pgp either in GB or BBB represents a double obstacle for the success of chemotherapy. Notwithstanding different approaches to circumvent the Pgp efflux activity of BBB [9,10,11,12,13,14] and GB, in particular of GB SCs [15,16,17], no satisfactory tools have been found.

Our research group has recently developed a library of Pgp ligands, based on the tetrahydroisoquinoline scaffold, a substructure characterizing several Pgp ligands [18,19]. The compounds were designed by functionalizing the phenolic group of **MC70**, an already known Pgp inhibitor [20] with two types of substituents: 1,2,5-oxadiazole (furazan) moiety linked through alkyl spacers [18], and flexible alkyl chains of different length [19]. From this library, we selected 6 compounds with an EC_50_ for Pgp ranging from 0.60 nM to 54 nM (Table 1), i.e., superimposable with the last-generation of Pgp inhibitors [21].

We investigated whether these compounds overcame the Pgp activity in BBB and GB cells, using human brain microvascular endothelial cells and GB cells obtained from patients, isolated and propagated as differentiated (adherent cell, AC) or stem cell-enriched (neurospheres, NS) cultures. In isolated BBB and GB, as well as in co-culture systems, we studied the effects of Pgp ligands on the transport and accumulation on doxorubicin, chosen as a prototypical drug that does not cross BBB [12,22] and is ineffective against GB NS [16], being a substrate of Pgp.

## 2. Results

### 2.1. Chemistry

**Scheme 1:**
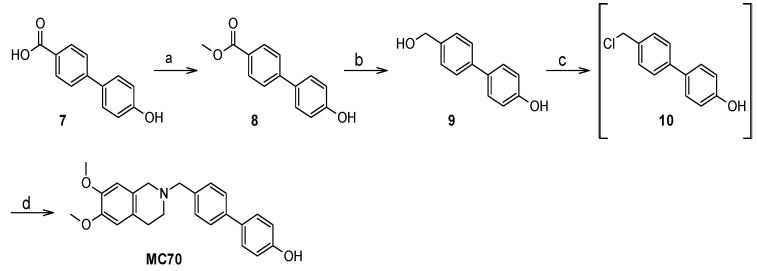
Synthesis of **MC70**. *Reagents and conditions*. (**a**) CH_3_OH, catatlytic concentrated H_2_SO_4_, reflux, 90 min; (**b**) LiAlH_4_, tetrahydrofuran, room temperature 45 min; (**c**) HCl 37%, 90 °C, 2 h; (**d**) 6,7-dimethoxy-1,2,3,4-tetrahydroisoquinoline hydrochloride, 4-methylmorpholine, CH_3_CN, reflux, 6 h.

Compounds **1**–**5** [18] and **6** [19] were synthesized from **MC70**, which was prepared according to the straightforward metal-free synthetic route reported in Scheme 1.

Briefly, the commercially available 4′-hydroxybiphenyl-4-carboxylic acid **7** was converted into the corresponding methyl ester by refluxing in methanol, in the presence of a catalytic amount of concentrated sulfuric acid. The product was reduced to the benzyl alcohol **9** in presence of LiAlH_4_ at room temperature. Treatment of **9** with 37% HCl at 90 °C afforded the benzyl chloride **10** which reacted with 6,7-dimethoxy-1,2,3,4-tetrahydroisoquinoline hydrochloride to give **MC70**.

### 2.2. Pgp Ligands Increase the Delivery of Doxorubicin Across BBB Monolayer

**Figure 1:**
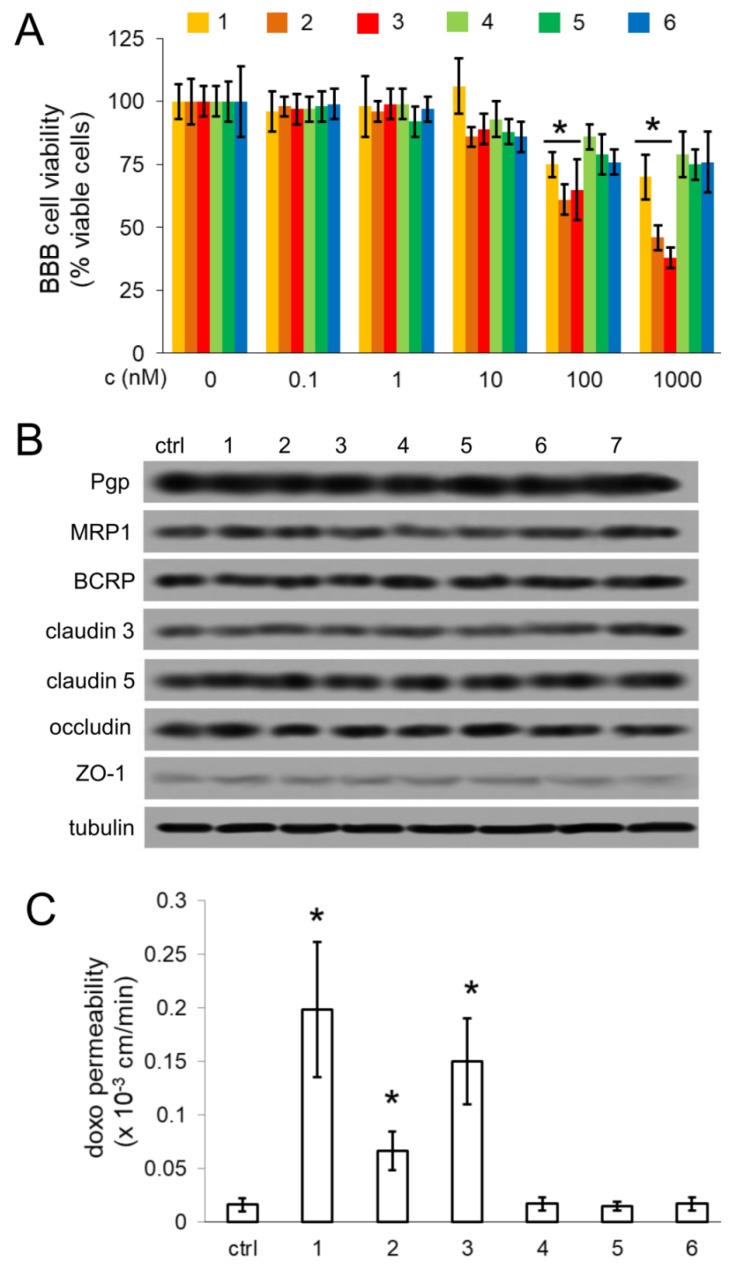
Effects of Pgp ligands of BBB viability and integrity. (A) Human cellular microvascular endothelial cells/D3 clone hCMEC/D3 cells were grown in the upper insert of Transwell devices for 7 days. The medium was then replaced with fresh medium (0) or with medium containing compounds **1**–**6** at the indicated concentrations for 24 h. Cell viability was measured by a chemiluminescence-based assay, in triplicates. Data are presented as means ± SD (*n* = 4). Versus untreated (0) cells: * *p* < 0.05. (**B**) hCMEC/D3 cells were grown in the upper insert of Transwell devices for 7 days. The medium was then replaced with fresh medium (ctrl) or with medium containing 1 nM of compounds **1**–**7** for 24 h. Cells were lysed and immunoblotted with the indicated antibodies. β-Tubulin level was used as control of equal protein loading. The figure is representative of one out of three experiments with similar results. (**C**) Cells were grown in the upper insert of Transwell devices and incubated as indicated in **B**. 5 μM doxorubicin (doxo) was added during the last 3 h. The amount of doxorubicin in the medium of the lower chamber was measured spectrofluorimetrically, in duplicates. Data are presented as means ± SD (*n* = 4). Versus dox: * *p* < 0.005.

In preliminary dose-dependence experiments, we verified the lack of toxicity of the compounds on the brain human cellular microvascular endothelial cells/D3 clone (hCMEC/D3) monolayer. BBB cell viability was significantly reduced after 24 h of incubation with compounds **1**–**3** at 100 and 1000 nM. None of the compounds decreased cell viability at 1 nM (Figure 1A), a concentration around their EC_50_ on Pgp [18,19] that was chosen for the following experiments. At this concentration the compounds did not change the expression of the ABC transporters Pgp, MRP1 and BCRP nor of TJ proteins claudin 3, claudin 5, occludin and zonula occludens-1 (ZO-1; Figure 1B). Compounds **1**–**3**—but not compounds **4**–**6**—significantly increased the delivery of doxorubicin, a virtually BBB-impermeable drug [12,22], through hCMEC/D3 cells monolayer (Figure 1C). By contrast, the permeability of mitoxantrone, an index of BCRP activity [12], was not affected (Supplementary Figure S1). On the other hand, doxorubicin is also a substrate of MRP1 and BCRP. Consistently, doxorubicin delivery across hCMEC/D3 cells monolayer was increased by the MRP1 inhibitor MK571 or by the BCRP inhibitor fumitremorgin C (Supplementary Figure S2), but MRP1 and BCRP inhibitors did not modify the effects of compounds **1**–**6** (Supplementary Figure S2). This may sound surprising since MK571 and fumitremorgin C alone increased the permeability of doxorubicin across hCMEC/D3 cells monolayer (Supplementary Figure S2). To clarify this issue, we tested the permeability of doxorubicin in Madin-Darby Canine Kidney (MDCK) cells selectively overexpressing Pgp, MRP1 or BCRP (Supplementary Figure S3A), treated with compounds **1**–**6**. As shown in the new Supplementary Figure S3B–D, compounds **1**–**3** increased doxorubicin permeability across Pgp-MDCK monolayer, but reduced the drug transport across MRP1-MDCK or BCRP-MDCK monolayer. 

This result is suggestive of the activation of MRP1 and BCRP, thus explaining why compounds **1**–**3** did not increase doxorubicin transport **3** in the presence of MRP1 or BCRP inhibitors. As expected, the Pgp inhibitor verapamil increased doxorubicin permeability in untreated Pgp-MDCK cells and in cells treated with the Pgp ligands: the extent of such increase was higher in cells exposed to compounds **1**, **2** and **3**, and similar to the control in cells treated with compounds **4**, **5** and **6** (Supplementary Figure S3B). On the one hand, compounds **1**, **2** and **3** slightly reduced doxorubicin delivery across MRP1-MDCK (Supplementary Figure S3C) and BCRP-MDCK (Supplementary Figure S3D) cells. On the other hand, compounds **4**, **5** and **6**, which did not change doxorubicin transport across Pgp-MDCK cells, except in the presence of verapamil, strongly reduced doxorubicin transport across MRP1-MDCK and BCRP-MDCK cells. These effects were reversed by MK571 and fumitremorgin C, respectively (Supplementary Figure S3B–D). The transendothelial electrical resistance (TEER) value of BBB monolayer was between 28 and 38 Ω cm^2^, the permeability coefficient of 70-kDa dextran-fluorescein isothiocyanate (FITC), an index of TJs integrity [23] was 0.21 ± 0.05 × 10^−3^ cm·min^−1^, the permeability coefficients of [^14^C]-sucrose, [^14^C]-inulin and lucifer yellow, indexes of paracellular diffusion [22,23,24] were 1.28 ± 0.19 × 10^−3^ cm·min^−1^, 0.45 ± 0.07 × 10^−3^ cm·min^−1^ and 0.43 ± 0.11 × 10^−3^ cm·min^−1^. These values were in line with previous findings [12,22,23,24], suggesting the functional integrity of the BBB monolayer. None of the compounds changed the TEER of BBB monolayer a 1 nM, while at 100 nM compounds **1**–**3** decreased TEER values indicating the loss of BBB integrity (Table 2). When used at 1 nM concentration, none of the compounds changed the permeability of 70-kDa dextran, [^14^C]-inulin, [^14^C]-sucrose and lucifer yellow (Supplementary Figure S4A–D).

### 2.3. Pgp Ligands Increase Doxorubicin Uptake and Cytotoxicity in Pgp-Positive Neurosphere of Glioblastoma

**Figure 2:**
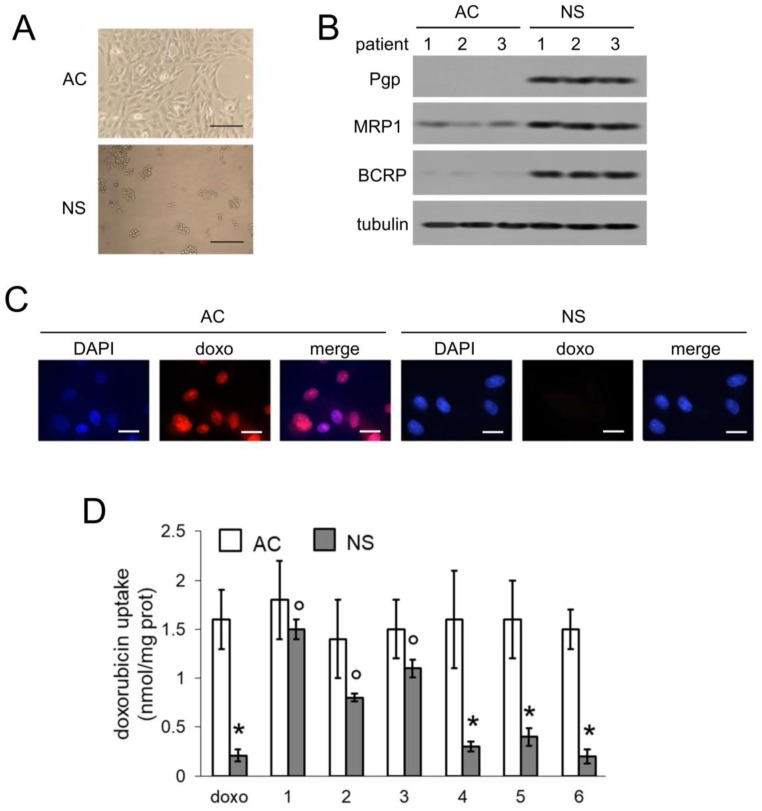
Effects of Pgp ligands on doxorubicin retention in glioblastoma cells. (**A**) Representative bright field microscope images of glioblastoma cells, cultured as adherent cells (AC) or neurospheres (NS). Magnification: 60 × objective (0.52 numerical aperture); 10 × ocular lens. Bar: 20 µm. The micrographs are representative of patient 2. No significant differences in cell morphology were detected for patient 1 and 3. (**B**) AC or NS from each patient were lysed and immunoblotted with the indicated antibodies. β-Tubulin level was used as control of equal protein loading. The figure is representative of one out of three experiments with similar results. (**C**) AC and NS from patient 2 were seeded on sterile glass coverslips, incubated 3 h with 5 μM doxorubicin (doxo), then stained with 4′,6-diamidin-2-phenylindole (DAPI) and analyzed by fluorescence microscopy to detect the intracellular accumulation of the drug. Magnification: 63 × objective (1.4 numerical aperture); 10 × ocular lens. The micrographs are representative of three experiments with similar results. No significant differences were detected for patient 1 and 3. Bar: 5 µm. (**D**) AC and NS were incubated for 3 h with 5 µM doxorubicin (doxo), in the absence or presence of 1 nM of compounds **1**–**6**. The intracellular doxorubicin was quantified fluorimetrically, in duplicates. Pooled data of patients 1–3 are presented as means ± SD (*n* = 3). Vs AC doxo: * *p* < 0.001; vs. NS doxo: ° *p* < 0.001.

We next validated the efficacy of our compounds against primary GB cells of 3 patients. From each tumor, AC and NS were generated (Figure 2A). As previously shown [16], NS had typical stemness properties, such as self-renewal, in vitro clonogenicity and in vivo tumorigenicity. In parallel, NS had high expression of general and neural stemness markers (nestin, CD133, Musashi, SOX2, EGFR, p53) and low expression of differentiation markers (glial fibrillary acidic protein, GFAP; galactocerebroside-C, Gal-C) compared to AC (Supplementary Table S1). This phenotype is indicative of a culture enriched in GB-derived SCs. As shown in Figure 2B, AC had undetectable levels of Pgp and low levels of MRP1 and BCRP. By contrast, all these ABC transporters were well-detected in the corresponding NS. In keeping with this trend, fluorescence microscope analysis revealed a detectable uptake of doxorubicin, that had a nuclear localization, in AC. In keeping with the higher ABC transporters expression, NS had no appreciable red fluorescence, indicating a very low drug uptake (Figure 2C). This difference was confirmed by the fluorimetric quantification of doxorubicin in AC and NS (Figure 2D). NS, which had a lower intracellular retention of the drug compared to AC, significantly increased doxorubicin accumulation if treated with compounds **1**–**3**. Compounds **4**–**6** had no effects. Moreover, none of the compounds increased the drug uptake in AC compared to untreated cells (Figure 2D).

**Figure 3:**
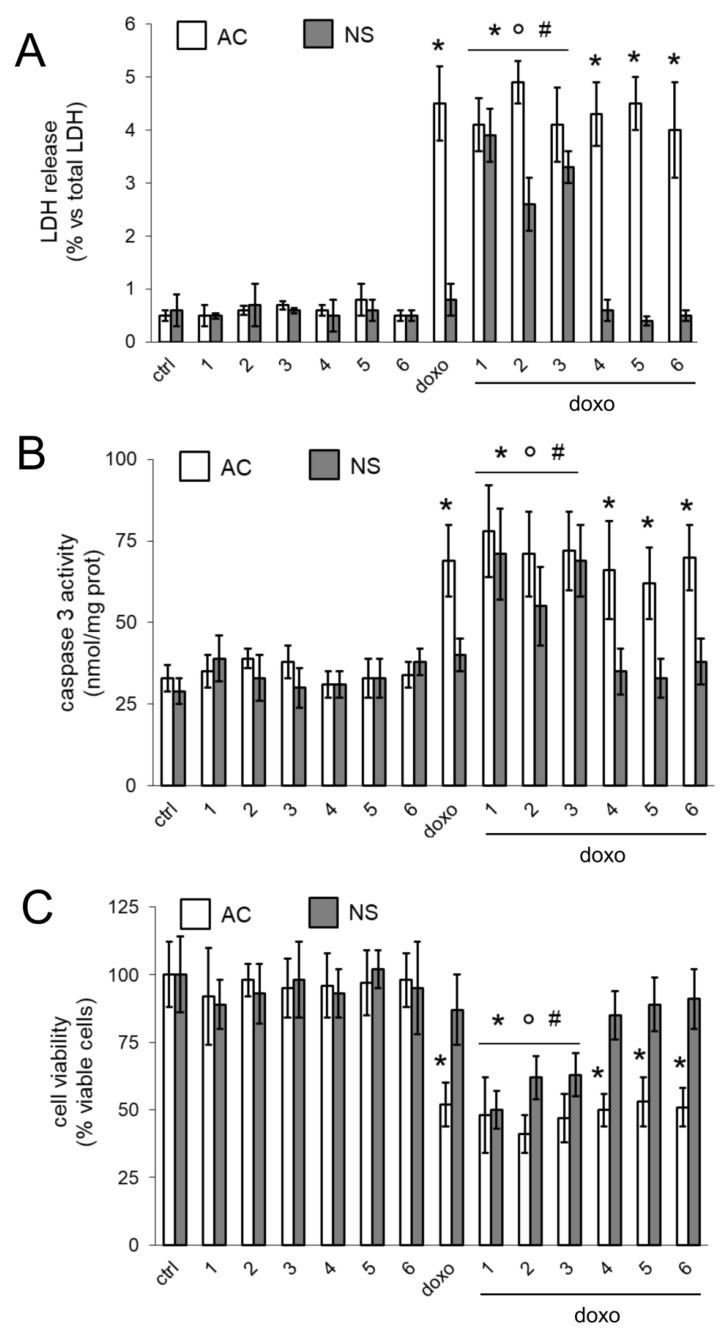
Effects of Pgp ligands on doxorubicin cytotoxicity in glioblastoma cells. Adherent cells (AC) or neurospheres (NS) from glioblastoma samples were grown for 24 h (panel **A**–**B**) or 48 h (panel **C**) in fresh medium (ctrl) or in the presence of 1 nM of compounds **1**–**6**. When indicated, 5 µM doxorubicin (doxo) was co-incubated. (**A**) The cell culture supernatant was checked spectrophotometrically for the extracellular activity of LDH, in duplicates. Pooled data of patients 1-3 are presented as means ± SD (*n* = 3). Vs. AC ctrl: * *p* < 0.001; vs. NS ctrl: ° *p* < 0.001; vs. NS doxo: ^#^ *p* < 0.001. (**B**) The activity of caspase 3 was measured fluorimetrically, in duplicates. Data are means ± SD (*n* = 3). Pooled data of patients 1–3 are presented as means ± SD (*n* = 3). Vs. AC ctrl: * *p* < 0.001; vs. NS ctrl: ° *p* < 0.01; vs. NS doxo: ^#^ *p* < 0.05. (**C**) Cell viability was measured by a chemiluminescence-based assay, in quadruplicates. Pooled data of patients 1-3 are presented as means ± SD (*n* = 3). Vs. AC ctrl: * *p* < 0.001; vs. NS ctrl: ° *p* < 0.001; vs. NS doxo: ^#^ *p* < 0.005.

Pgp ligands were not toxic on GB cells: indeed, they did not increase the release of lactate dehydrogenase (LDH; Figure 3A), and index of cell damage and necrosis [16], they did not activate caspase 3 (Figure 3B), an index of apoptosis, and they did not reduce AC and NS viability (Figure 3C). According to its differential intracellular retention, doxorubicin increased LDH release and caspase 3 activity, and decreased cell viability in AC but not in NS. Compounds **1**–**3** partially restored doxorubicin’s cytotoxic effects in NS. Again the compounds did not enhance the anthracycline’s effects in AC. Compounds **4**–**6**—that did not increase doxorubicin accumulation in NS (Figure 2D)—did not restore the drug’s toxicity as well (Figure 3A–C). Pgp ligands increase the intra-tumor delivery and cytotoxicity of doxorubicin in BBB-glioblastoma co-cultures.

**Figure 4:**
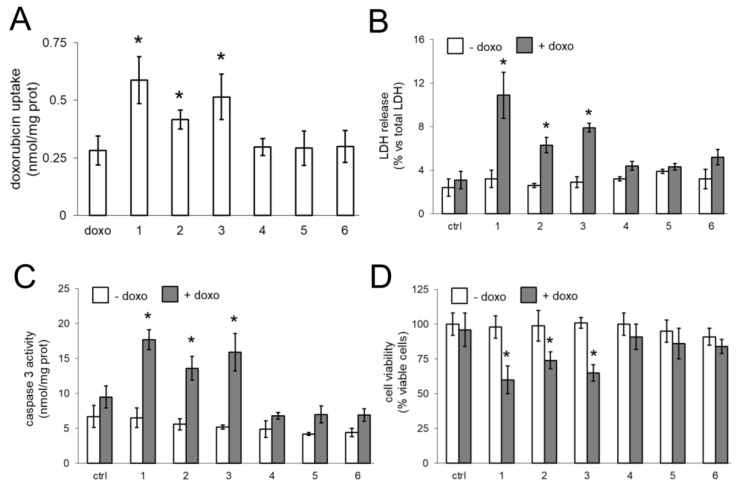
Effects of Pgp ligands on doxorubicin delivery and cytotoxicity in BBB-glioblastoma cells co-cultures. hCMEC/D3 cells were grown for 7 days up to confluence in Transwell inserts; neurospheres (NS) were seeded at day 4 in the lower chamber. After 3 days of co-culture, supernatant in the upper chamber was replaced with fresh medium (ctrl) or with medium containing 5 µM doxorubicin (doxo), in the absence (ctrl) or presence of 1 nM of compounds **1**–**6**. (**A**) Fluorimetric quantification of intracellular doxorubicin in NS after 6 h. Pooled data of patients 1-3 are presented as means ± SD (*n* = 3). Vs. doxo: * *p* < 0.01. (**B**) The culture supernatant of NS was checked spectrophotometrically for the extracellular activity of LDH after 24 h. Pooled data of patients 1–3 are presented as means ± SD (*n* = 3). Vs. untreated cells (ctrl, either “−doxo” or “+doxo”): * *p* < 0.001. (**C**) The activity of caspase 3 was measured fluorimetrically in NS lysates after 24 h, in duplicates. Pooled data of patients 1–3 are presented as means ± SD (*n* = 3). Vs. untreated cells (ctrl, either “−doxo” or “+doxo”): * *p* < 0.002. (**D**) Cell viability of NS was measured after 48 h by a chemiluminescence-based assay, in quadruplicates. Pooled data of patients 1–3 are presented as means ± SD (*n* = 3). Vs. untreated cells (ctrl, either “−doxo” or “+doxo”): * *p* < 0.02.

We finally validated the efficacy of our Pgp ligands in co-culture systems: doxorubicin-resistant NS were seeded in the lower chamber of Transwell devices, containing confluent hCMEC/D3 monolayer in the upper chamber. Doxorubicin, alone or co-incubated with the compounds, was added in the upper chamber, facing the luminal side of BBB cells. In these conditions, the amount of doxorubicin delivered into NS was low (Figure 4A) and unable to elicit cell necrosis (Figure 4B), apoptosis (Figure 4C) or reduction in NS viability (Figure 4D). The co-incubation with compounds **1**–**3** significantly increased the amount of doxorubicin delivered to NS (Figure 4A), the release of LDH (Figure 4B) and the activity of caspase 3 (Figure 4C) after 24 h. The co-incubation of doxorubicin and compounds **1**–**3** also reduced NS viability after 72 h (Figure 4D).

**Figure 5:**
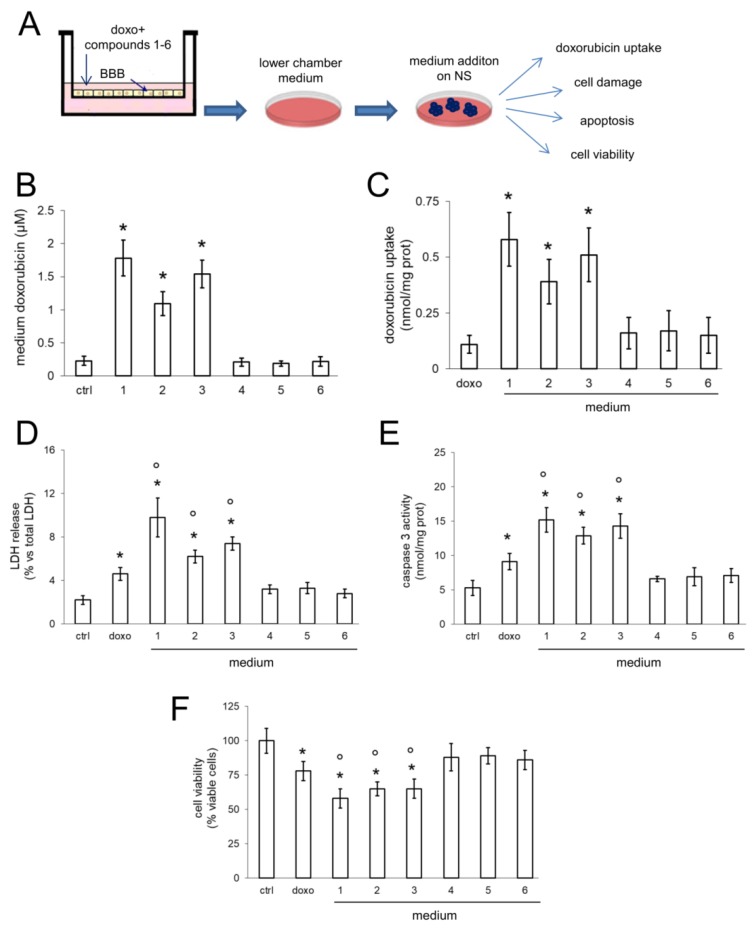
Possible dual effects of Pgp ligands on Pgp in BBB and glioblastoma cells. (**A**) hCMEC/D3 cells were grown for 7 days up to confluence in Transwell inserts, then the supernatant in the upper chamber was replaced with fresh medium (ctrl) or with medium containing 5 µM doxorubicin, in the absence (ctrl) or presence of 1 nM of compounds **1**–**6**. After 3 h the medium of the lower chamber was removed and added to neuropsheres (NS) for the measure of intracellular doxorubicin uptake, LDH release, caspase-3 activity and cell viability (panels **C**–**F**). In all these panels, a standard solution of 1 µM doxorubicin (doxo) was used as internal control. (**B**) Fluorimetric quantification of doxorubicin in the medium of the lower chamber incubated as reported in **A**. Data are means ± SD (*n* = 3). Vs ctrl: * *p* < 0.001. (**C**) Fluorimetric quantification of intracellular doxorubicin in NS after 6 h, treated with 1 µM doxorubicin (doxo) or with media of the lower chamber containing 5 µM doxorubicin+compounds **1**–**6**. Pooled data of patients 1–3 are presented as means ± SD (*n* = 3). Vs. doxo: * *p* < 0.001. (**D**) The culture supernatant of NS, treated as in **C** was checked spectrophotometrically for the extracellular activity of LDH after 24 h. Pooled data of patients 1-3 are presented as means ± SD (*n* = 3). Vs. untreated cells (ctrl): * *p* < 0.005; vs. cells treated with 1 µM doxorubicin (doxo): * *p* < 0.02. (**E**) The activity of caspase 3 was measured fluorimetrically in NS lysates after 24 h of treatment as in **C** in duplicates. Pooled data of patients 1–3 are presented as means ± SD (*n* = 3). Vs. untreated cells (ctrl): * *p* < 0.02; vs. cells treated with 1 µM doxorubicin (doxo): * *p* < 0.02. (**F**) Cell viability of NS, treated as in **C**, was measured after 48 h by a chemiluminescence-based assay, in quadruplicates. Pooled data of patients 1–3 are presented as means ± SD (*n* = 3). Vs. untreated cells (ctrl): * *p* < 0.05; vs. cells treated with 1 µM doxorubicin (doxo): * *p* < 0.05.

To discriminate whether the effects of the Pgp ligands were solely due to the inhibition of Pgp on BBB, or to their inhibitory effects on Pgp both on BBB and NS (i.e., after crossing the barrier), we added 5 μM doxorubicin, alone or with compounds 1–6, in the upper chamber of Transwell devices containing BBB. After 3 h, the medium of the lower chamber was collected: part was added to NS cultures (Figure 5A), part was used to measure the doxorubicin concentration (Figure 5B). The drug concentration in the media of the lower chamber ranged between 1 and 1.8 μM for the Transwells treated with compounds 1–3, and was significantly higher than in all the other experimental conditions (Figure 5B). The effects elicited by the media derived from these Transwells was compared with the effects produced by medium containing 1 μM doxorubicin. Of note, the intracellular doxorubicin uptake in NS (Figure 5C), the release of LDH (Figure 5D), the activity of caspase-3 (Figure 5E) were higher, whereas the viability was lower (Figure 5F) when NS were treated with media derived from Transwells exposed to compounds 1–3 than with medium containing 1 μM doxorubicin. These results suggest a possible effect of the compounds on Pgp present either on BBB cells or NS.

## 3. Discussion

Many Pgp inhibitors that have displayed excellent in vitro efficacy have failed in pre-clinical and clinical models because of their great toxicity, owing to the high (i.e., millimolar-micromolar) concentrations required to inhibit Pgp, which results in heavy side-effects and toxicities [25]. The research for more effective P-glycoprotein inhibitors unveiled that Pgp-expressing tumor cells retain sensitivity to local anaesthetics, detergents, antimetabolites, alkylating agents, platinum compounds, metal chelators. These findings opened the opportunity to bypass MDR by treating Pgp-expressing cells with non-cross-resistant drugs, exploiting the peculiar sensitivity (called “collateral sensitivity”) of resistant cells to these agents [26]. Despite the promising results obtained in vitro, the in vivo safety of these agents, most of which exert cytotoxic effects in cell cultures, is not known.

Targeting ABC efflux transporters with new chemosensitizers is still considered the main strategy to improve drug delivery and overcome MDR [27], but it remains an unmet need. Compared to the first Pgp inhibitors, the latest generation of Pgp inhibitors, such as tariquidar, elacridar or zosuquidar, showed efficacy at lower concentrations (i.e., nanomolar concentrations) and higher specificity for Pgp over the other ABC transporters [21].

Furazan-based compounds **1**–**5** were originally designed following the experimental observation that specific furazan derivatives inhibited Pgp (unpublished data). In the series presented in this work, the stereo-electronic properties of the substituents on the heterocyclic ring were modulated. Compound **6** belongs to a series of derivatives designed to study the effects of lipophilicity of the substituents on the phenolic group of parental compound. According to the EC_50_ values, the selected **MC70** derivatives were more potent and displayed a functional profile different compared to **MC70**: the latter behaves as a Pgp inhibitor, since it has an apparent permeability ratio < 2, determined in Caco-2 cells monolayer, and does not induce ATP depletion; the new compounds are substrates belonging to the class IIB3 of Pgp substrates, characterized by apparent permeability > 2 and absence of ATP depletion [18,19]. Kinetic parameters for these derivatives have not been determined, but, as a cautious consideration, it can be argued that, due to the large internal cavity of Pgp, the binding of these ligands could be approximately considered only diffusion-limited; in such cases, a typical residence time for nanomolar ligands would be around 1 s [28].

Three out of six compounds, namely **1**–**3**, effectively increased the transport of doxorubicin, a virtually BBB-impermeable drug, being a substrate of Pgp [7], across BBB monolayer, when used at 1 nM concentration. Notably, at such concentration they did not reduce BBB cell viability and they did not change the expression of other luminal ABC transporters and TJ proteins, and the TEER values. This experimental set demonstrated that at 1 nM concentration the compounds do not compromise the integrity and the physiological properties of BBB. Of note, compounds **1**–**3** strongly inhibited Pgp activity, but slightly activated MRP1 and BCRP, as demonstrated by the increased transport of doxorubicin across Pgp-MDCK monolayer, by the decreased transport across MRP1-MDCK and BCRP-MDCK monolayer, by the lack of increase in doxorubicin transport across hCMEC/D3 monolayer treated with MRP1 and BCRP inhibitors compared to untreated cells. These contrasting effects may reduce the efficacy of compounds **1**–**3** on doxorubicin transport: on the one hand the compounds increased the drug delivery by inhibiting Pgp, on the other hand they decreased the drug transport by stimulating MRP1 and BCRP. The activating effect on MRP1 and BCRP, however, was smaller compared to the inhibitory effect on Pgp, in terms of doxorubicin transport, as demonstrated by the results in MDCK cells selectively expressing Pgp, MRP1 or BCRP. Therein, the net effect in hCMEC/D3 cells, where these three transporters were present, was a significant increase of the transport of doxorubicin across BBB monolayer. Compounds **4**, **5** and **6** did not affect doxorubicin transport across Pgp-MDCK cells, where MRP1 and BCRP levels were undetectable, but they strongly reduced the drug transport in MRP1- and BCRP-MDCK cells, where Pgp was undetectable. These results suggest that compounds **4**, **5** and **6** were likely activators of MRP1 and BCRP, but the activating effect was reversed by selective MRP1 and BCRP inhibitors According to the results obtained with compounds **1**–**3** (inhibitors of doxorubicin transport by Pgp, slight activators of doxorubicin transport by MRP1 and BCRP) and with compounds **4**–**6** (neither inhibitors nor activators of doxorubicin transport by Pgp, strong activators of doxorubicin transport by MRP1 and BCRP), we can hypothesize that in hCMEC/D3 cells the main transporter involved in doxorubicin transport is Pgp, while MRP1 and BCRP play an ancillary role. We hypothesize that this could be the reason why we did not detect any effect of compounds **4**–**6** on doxorubicin delivery across BBB monolayer. We are aware that all the compounds exerted an inhibition on Pgp activity, measured as calcein acetoxymethyl ester (AM) transport (Table 1). Given the complex structure of Pgp, containing different drug binding sites and characterized by different affinity for different substrates, it is not surprising that compounds inhibiting the transport of one substrate, have no effect or even opposite effects on the transport of other substrates [29]. Since our aim was to obtain a net increase in doxorubicin delivery across BBB to make it an effective anti-GB drug, the most promising compounds in this perspective were **1**, **2** and **3**.

All the compounds did not change the permeability of high molecular weight (70-kDa dextran) or low molecular weight ([^14^C]-inulin, [^14^C]-sucrose, lucifer yellow) compounds. Therefore, we excluded that the effects on doxorubicin permeability was due to the loss of TJs integrity and to a possible paracellular diffusion of the drug across BBB.

Overall, our compounds showed the same properties—i.e., efficacy at low nanomolar concentrations, specificity for Pgp and lack of in vitro toxicities on not-transformed cells—of the latest generation of Pgp inhibitors or tracers, under investigation in preclinical models and clinical trials [10,14,21].

The selectivity for Pgp was demonstrated also in the experiments performed on AC and NS generated from patients GB. While AC where low Pgp-expressing cells and retained high amount of doxorubicin, NS were chosen as a prototypical model of highly Pgp-expressing GB-derived SCs [16]. We recognize that NS do not reproduce the tissue organization and cell polarity occurring in vivo in the microvascular niches, that include inter-connected endothelial cells, pericytes, astroglial and microglial cells, differentiated GS cells and GB SCs, actively proliferating and apoptotic/necrotic GB cells. However, NS have been proven to be a reliable tool to measure the chemosensitizing efficacy of Pgp-reversing agents in GB and BBB-GB co-cultures [12,13,16]. We thus compared the effects of our compounds on AC and NS, alone or growing under hCMEC/D3 monolayer. The compounds did not exert any additive effects to doxorubicin in AC, where Pgp was undetectable. In AC, doxorubicin likely reached its maximal accumulation and exerted a broadest spectrum of cytotoxic effects, including increased cell necrosis, increased poptosis and reduced viability. By contrast, the presence of Pgp in NS limited the retention and cytotoxic efficacy of doxorubicin. In NS compounds **1**–**3** restored the drug’s accumulation to the same levels of AC, suggesting that they inhibited the drug efflux via Pgp. As already observed in BBB, the compounds alone were not toxic against GB cells. Only the association of compounds and doxorubicin induced cytotoxicity, suggesting that the compounds acted as chemosensitizers agents, rescuing the efficacy of doxorubicin.

We could not directly demonstrate the transport of compounds **1**–**3** across BBB, since nanomolar concentrations were below the detection limit of our high-pressure liquid chromatography (HPLC) device and micromolar concentrations, that were well-measured by HPLC, reduced cell viability of BBB, suggesting that at these concentrations BBB is damaged and does not represent a physiologically competent BBB. However, in co-culture experiments, when we exposed the luminal side of BBB with the compounds **1**–**3** associated with doxorubicin, we observed increased delivery and cytotoxicity of doxorubicin in NS growing under competent BBB. In BBB monolayer, Pgp is expressed on the luminal side [30], i.e., facing doxorubicin. The increased efficacy of doxorubicin may be due to a simple increase in the drug delivery across BBB, consequent to the inhibition of Pgp present on BBB. However, also NS express Pgp. According to the low intracellular retention of doxorubicin within NS, it is likely that Pgp is expressed on the outer surface of the spheres, preventing the intracellular accumulation of the drug.

Therein, doxorubicin delivered across BBB could not be efficiently accumulated within NS nor exert its cytotoxic effects if the Pgp present in NS was active. The results with media collected from the lower chamber of Transwell devices incubated with compounds **1**–**3** suggested that the compounds inhibit at the same time Pgp on BBB and NS cells: this could be an indirect evidence of the delivery of compounds **1**–**3** across BBB. We are currently testing this hypothesis evaluating the pharmacokinetic profile of the compounds systemically administered in mice bearing orthotopically implanted GB.

Notwithstanding the excellent anti-tumor activity against GB in in vitro [31], doxorubicin is not a first-choice drug in GB treatment because of the very low delivery across BBB. However, since GB AC are sensitive to doxorubicin [31], several strategies to improve the in vivo efficacy of doxorubicin are being investigated in pre-clinical and clinical settings [32,33,34]. Our compounds represent a step forward to this direction, since they transformed the BBB-impermeable doxorubicin into a drug with a good BBB permeability and efficacy against Pgp-positive NS.

Moreover, temozolomide, topoisomerase I and II inhibitors, that are the current first- and second-line therapies in GB, are substrates of Pgp [11,35]. Therein, this work may open the way to pre-clinical studies combining our Pgp ligands and these drugs, in order to circumvent Pgp-mediated chemoresistance and improve the efficacy of chemotherapy against GB.

## 4. Materials and Methods

### 4.1. Chemicals

The plasticware for cell cultures was obtained from Falcon (Becton Dickinson, Franklin Lakes, NJ, USA). The electrophoresis reagents were obtained from Bio-Rad Laboratories (Hercules, CA, USA). The protein content of cell lysates was assessed with the Bicinchoninic Acid (BCA) kit from Sigma Chemicals Co. (St. Louis, MO, USA). Unless specified otherwise, all reagents were purchased from Sigma Chemicals Co.

### 4.2. Synthesis and Characterization of Compounds

^1^H and ^13^C-NMR spectra were recorded on a Jeol 600 instrument (Jeol, Ltd., Tokyo, Japan) at 600 and 150 MHz, respectively, using SiMe_4_ as the internal reference. Chemical shifts (δ) are given in parts per million (ppm). The following abbreviations are used to designate the multiplicities: s = singlet, d = doublet, dd = doublet of doublets, t = triplet, q = quartet, m = multiplet. Low resolution mass spectra were recorded on a Micromass Quattro microTM API (Waters Corporation, Milford, MA, USA) with electrospray ionization. Melting points (mp) were determined with a capillary apparatus (Büchi 540). Flash column chromatography was performed on silica gel (Kieselgel 60, 230–400 mesh ASTM, Merck KGaA, Darmstadt, Germany). The progress of the reactions was followed by thin layer chromatography (TLC) on 5 × 20 cm plates with a layer thickness of 0.2 mm. The purity of target compounds was assessed by reverse phase (RP)-HPLC. Analyses were performed on a HP1100 chromatograph system (Agilent Technologies, Palo Alto, CA, USA). The analytical column was a LiChrosphere® C18 5µM (Merck KGaA).Ultraviolet UV signals were recorded at 210, 226 and 254 nm. All compounds were dissolved in eluent and injected through a 20 μL loop. Compounds **1**–**5** and **6** were synthesized as previously detailed [18,19].

*Methyl 4′-hydroxybiphenyl-4-carboxylate* (**8**). 4′-hydroxybiphenyl-4-carboxylic acid **7** was dissolved in methanol; to the solution 5 µL of concentrated sulfuric acid were added and the mixture was refluxed for 90 min. The white solid separated from the boiling mixture which was isolated by filtration. Yield: 78%. Mp = 231.1–231.5 °C. MS ESI^−^: 227 [M-1]^−^. ^1^H-NMR (DMSO-*d*_6_) δ 9.79 (s. 1H, C_6_H_4_OH), 7.97 (d, *J* = 8.3 Hz, 2H), 7.72 (d, *J* = 8.6 Hz, 2H), 7.58 (d, *J* = 8.6 Hz, 2H), 6.88 (d, *J* = 8.6 Hz, 2H), 3.85 (s, 3H, OCH_3_). ^13^C-NMR (DMSO-*d*_6_) δ166.16, 158.08, 144.73, 129.79, 129.40, 128.20, 127.31, 125.97, 115.94, 52.06.

*4′-(Hydroxymethyl)biphenyl-4-ol* (**9**). LiAlH_4_ (1.3 equivaments, eq.) was suspended in anhydrous tetrahydrofuran under N_2_ atmosphere. A solution of **8** in anhydrous tetrahydrofuran was added through a dropping funnel and the mixture was stirred at room temperature for 45 min. The mixture was then cooled in an ice bath and was quenched with ice-cold water and 1M HCl. The aqueous phase was extracted with ethyl acetate; the organic extracts were washed with brine, dried over Na_2_SO_4_, filtered and evaporated under reduced pressure to afford the title product as a white solid with 89% yield. Mp = 204.2–205.0 °C (decomposition.). MS ESI^−^: 227 [M-1]^−^. ^1^H-NMR (DMSO-*d*_6_) δ 9.54 (s. 1H, C_6_H_4_OH), 7.53 (d, *J* = 8.3 Hz, 2H), 7.47 (m, 2H), 7.35 (d, *J* = 8.3 Hz, 2H), 6.85 (m, 2H), 5.2 (t, *J* = 5.7 Hz, 1H, CH_2_OH), 4.52 (d, *J* = 5.9 Hz, 2H, CH_2_OH). ^13^C-NMR (DMSO-*d_6_*) δ157.02, 140.65, 138.68, 130.91, 127.64, 127.04, 125.68, 115.74, 67.2.

*[4′-(6,7-Dimethoxy-3,4-dihydro-1H-isoquinolin-2-ylmethyl)biphenyl-4-ol]* (**MC70**). Compound **9** was suspended in 37% HCl and the mixture was stirred at 90 °C for 2 h. The suspension was cooled in an ice bath, diluted with ice-cold water and filtered under reduced pressure to give **10** as a white solid. The latter, after being dried over KOH, was dissolved in acetonitrile. 6,7-dimethoxy-1,2,3,4-tetrahydroisoquinoline hydrochloride (1.3 eq.) and 4-methylmorpholine (2.3 eq) were added to the solution and the mixture was refluxed for 6 h. The solvent was then evaporated under reduced pressure, the residue was taken up with water and extracted with ethyl acetate. The organic extracts were washed with brine, dried over Na_2_SO_4_, filtered and evaporated under reduced pressure. The crude product was purified on silica gel column, eluting with petroleum ether/acetone 70/30, to give the title product identical to an authentic sample [19]. Yield was 56% over two steps.

The Pgp activity in the presence of the compounds was evaluated by the Calcein-AM assay and the bioluminescent ATP assay, as described previously [18,19].

### 4.3. BBB Cells, TEER and Permeability Assays

hCMEC/D3 cells, a human brain microvascular endothelial stabilized cell line, were a kind gift from Prof. Pierre-Olivier Couraud (Institut Cochin, Centre National de la Recherche Scientifique UMR 8104, INSERM U567, Paris, France) and were cultured according to [22]. Cells were seeded at 50,000/cm² density, and grown for 7 days up to confluence in 6-well Transwell devices (0.4 μm diameter pores-size, Transwell insert surface: 4.67 cm^2^; Corning Life Sciences, Chorges, France for transport assays) or 24-well Transwell devices (0.4 μm diameter pores-size, Transwell insert surface: 0.33 cm^2^; Corning Life Sciences, for TEER measurements), to allow the formation of a competent BBB. Before each experiment, TEER and permeability coefficients of 70 kDa-Dextran FITC, [^14^C]-sucrose (589 mCi/mmol; PerkinElmer, Waltham, MA, USA), [^14^C]-inulin (10 mCi/mmol; PerkinElmer) and lucifer yellow (Invitrogen Life Technology, Milano, Italy), were measured as previously described [12,22,23,24] in BBB cells in the absence of GB cells. TEER was measured using a Voltohmetro Millicell-ERS (Millipore, Bedford, MA, USA), according to the manufacturer’s instructions. The mean TEER value of the plastic insert in the absence of cells was 26.73 Ω cm^2^ (*n* = 8). This value was subtracted from each value obtained in the presence of the cells.

For transport assays, after 7 days of culture, the culture medium was replaced in both chambers. 2 μM 70 kDa dextran-FITC, 2 μCi/ml [^14^C]-sucrose, 2 μCi/ml [^14^C]-inulin, 100 μM lucifer yellow were added to the upper chamber of Transwell. After 3 h the medium in the lower chamber was collected. The amount of [^14^C]-sucrose and [^14^C]-inulin was measured using a Tri-Carb Liquid Scintillation Analyzer (PerkinElmer). Radioactivity was converted in nmol/cm^2^, using a calibration curve previously prepared. The radioactivity of the medium alone, considered as a blank, was subtracted from each measure.

The amount of 70 kDa dextran-FITC and lucifer yellow was measured fluorimetrically, using a Synergy HT microplate spectrofluorimeter (Bio-Tek Instruments, Winooski, VT, USA). Excitation and emission wavelengths were: 494 nm and 518 nm (70 kDa dextran-FITC); 430 nm and 540 nm (lucifer yellow). Fluorescence was converted in nmol/cm^2^, using a calibration curve previously set. The autofluorescence of the medium, considered as a blank, was subtracted from each measure. The permeability coefficients were calculated according to [36].

MDCK, Pgp-MDCK, MRP1-MDCK and BCRP-MDCK cells were a kind gift of Dr. Maria Alessandra Contino (Department of Pharmacy, University of Bari, Italy). Culture and seeding conditions for doxorubicin transport assay were carried out as reported in [37].

### 4.4. GB Cells

Primary human GB cells (01010627, CV17, Nov3, here identified as “patients 1, 2 and 3”) were obtained from surgical samples of patients, from the Neurosurgical Unit, Universities of Torino, Italy, and Neuro-Bio-Oncology Center, Vercelli, Italy. All subjects gave their informed consent for inclusion before they participated in the study. The study was conducted in accordance with the Declaration of Helsinki, and the protocol was approved by the Ethics Committee of University of Torino (ORTO11WNST). The histological diagnosis was performed according to World Health Organization (WHO) guidelines. Cells were cultured as adherent cells (AC) or neurospheres (NS) as previously described [38], with minor modifications [16]. Phenotypic characterization of differentiation and stemness markers, in vitro clonogenicity and self-renewal, in vivo tumorigenicity are detailed in [16]. Morphological analysis was performed with a bright field microscope (Leica Microsystems, Wetzlar, Germany). For phenotypic characterization, the following antibodies were used: anti-nestin (Millipore), anti-CD133 (Miltenyi Biotec, Bergisch Gladbach, Germany), anti-Musashi (Millipore), anti-SOX2 (R&D Systems, Minneapolis, MN, USA), anti-EGF (Cell Signaling Technology Inc, Danvers, MA, USA), anti-p53 (Dako, Glostrup, Denmark), anti-GFAP (Dako), anti-Gal-C (Millipore), followed by goat anti-rabbit FITC-conjugated Immunoglobulin (IgG) and rabbit anti-mouse tetramethyl rhodamine iso-thiocyanate (TRITC)-conjugated IgG antibodies. Nuclei were counterstained with DAPI. The observations were made by immunofluorescence on a Zeiss Axioskop microscope equipped with an AxioCam5MRSc and coupled to the imaging system AxixoVision (Release 4.5, Zeiss, Oberkochen, Germany), by using a 6 × oil immersion objective (1.4 numerical aperture) and 10 × ocular lens. For each experimental point, a minimum of 5 microscopic fields were examined.

In co-culture experiments, 500,000 GB cells were added in the lower chamber of Transwell devices, 4 days after seeding hCMEC/D3 cells in the Transwell insert. After 3 days of co-culture the medium of the upper and lower chamber was replaced, and cells were used for the experimental assays.

### 4.5. Cell Viability

Cell viability was evaluated by ATPLite kit (PerkinElmer), as per manufacturer’s instructions. The results were expressed as percentage of viable cells in each experimental condition versus untreated cells (considered 100%).

### 4.6. Immunoblotting

Cells were rinsed with ice-cold lysis buffer (50 mM, Tris, 10 mM EDTA, 1% *v*/*v* Triton-X100), supplemented with the protease inhibitor cocktail set III (80 μM aprotinin, 5 mM bestatin, 1.5 mM leupeptin, 1 mM pepstatin; Calbiochem, San Diego, CA, USA), 2 mM phenylmethylsulfonyl fluoride and 1 mM Na3VO4, then sonicated and centrifuged at 13,000× *g* for 10 min at 4 °C. Twenty μg of extracted proteins were subjected to SDS-PAGE and probed with the following antibodies: anti-Pgp (C219; Calbiochem), anti-MRP1 (MRPm5; Abcam, city, UK), anti-BCRP (M-70; Santa Cruz Biotechnology Inc., Santa Cruz, CA, USA), anti-claudin 3 (PA5-16867; ThermoFisher Scientific, Waltham, MA, USA), anti-claudin 5 (4C3C2; ThermoFisher Scientific), anti-occludin (6HCLC; ThermoFisher Scientific), anti-ZO-1 (40-2200; ThermoFisher Scientific), anti-β-tubulin (D-10 and TUJ1; Santa Cruz Biotechnology Inc.), followed by a peroxidase-conjugated secondary antibody (Bio-Rad Laboratories). The membranes were washed with Tris-buffered saline-Tween 0.1% *v*/*v* solution, and the proteins were detected by enhanced chemiluminescence (Bio-Rad Laboratories).

### 4.7. Fluorescence Microscopy

GB cells were seeded on sterile glass coverslips and incubated 3 h with 5 µM doxorubicin, rinsed with phosphate-buffered saline (PBS) solution, fixed with 4% *w*/*v* paraformaldehyde for 15 min, washed three times with PBS and incubated with DAPI for 3 min at room temperature in the dark. Cells were washed three times with PBS and once with water, then the slides were mounted with 4 μL of Gel Mount Aqueous Mounting and examined with a Leica DC100 fluorescence microscope (Leica Microsystems GmbH). For each experimental point, a minimum of five microscopic fields were examined.

### 4.8. Doxorubicin Uptake

hCMEC/D3 cells, grown up to confluence for 7 days in Transwell devices, or GB cells were incubated 3 h with 5 µM doxorubicin, washed with PBS, trypsinized, centrifuged at 13,000× *g* for 5 min and re-suspended in 0.5 mL of 1/1 solution ethanol/0.3 N HCl. A 50 µL aliquot was sonicated and used for the measurement of the protein content. The intracellular fluorescence of doxorubicin was measured spectrofluorimetrically, using a Synergy HT microplate spectrofluorimeter (Bio-Tek Instruments). Excitation and emission wavelengths were 475 nm and 553 nm. Fluorescence was converted in nmol/mg cell proteins, using a calibration curve previously set. The intratumor doxorubicin delivery to GB grown under BBB monolayer was measured as previously described [11]. After 3 days of co-culture, 5 µM doxorubicin, alone or in the presence of the compounds, was added to the upper chamber of Transwell inserts containing hCMEC/D3 cells monolayer. After 3 h, GB cells were collected from the lower chamber and the intracellular amount of doxorubicin was measured spectrofluorimetrically as described above.

### 4.9. Cytotoxicity

The release of LDH in cell supernatant was measured as reported in [16], using a Synergy HT microplate reader. Both intracellular and extracellular enzyme activities were expressed as μmol NADH oxidized/min/dish, then extracellular LDH activity was calculated as percentage of the total LDH activity. For cytotoxicity assays in co-cultures, 5 μM doxorubicin, alone or in the presence of compounds, was added to the upper chamber of Transwell inserts. After 24 h, both cell culture medium and GB cells from the lower chamber were collected, and checked for the activity of LDH, as described above.

### 4.10. Caspase 3 Activity

The activity of caspase 3, taken as an index of apoptosis, was measured by incubating 20 µg of cell lysates, collected from GB cells or GB cells growing under BBB monolayer, as reported above, with the fluorogenic substrate of caspase 3 DEVD-7-amino-4-methylcumarine (DEVD-AMC), as reported in [11]. Results were expressed as nmoles AMC/mg proteins, using a calibration curve previously set.

### 4.11. Statistical Analysis

All data in the text and figures are provided as means ± SD. The results were analyzed by a one-way analysis of variance (ANOVA) and Tukey’s test. *p* < 0.05 was considered significant.

## Figures and Tables

**Table 1:** Structures and EC_50_ values of the tested Pgp ligands.

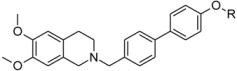		
Compound	R	EC_50_ (nM)
**1**	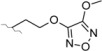	0.60 [18]
**2**	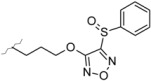	1.3 [18]
**3**	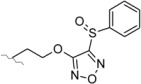	0.90 [18]
**4**	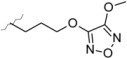	54 [18]
**5**	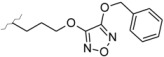	0.97 [18]
**6**	*n*-(C_4_H_9_)-	5.2 [19]
**7 (MC70)**	-H	690 [20]

**Table 2:** Transendothelial electrical resistance (TEER) values of BBB monolayer treated with Pgp ligands.

Compound	TEER (Ω cm^2^) Compound 1 nM	TEER (Ω cm^2^) Compound 100 nM
**Control**	31 ± 2	30 ± 3
**1**	33 ± 3	41 ± 2 *
**2**	29 ± 1	39 + 2 *
**3**	35 ± 3	44 ± 4 *
**4**	34 ± 4	33 ± 3
**5**	36 ±3	34 ±5
**6**	33 ± 2	31 ± 4

hCMEC/D3 cells were grown in the upper insert of Transwell devices for 7 days. The medium was replaced with fresh medium (control) or with medium containing 1 nM or 100 nM of compounds **1**–**6** for 24 h. TEER was measured in duplicates. 100 nM vs. 1 nM: * *p* < 0.05. Data are presented as means ± SD (*n* = 4). TEER values were subtracted from the mean TEER value obtained in the absence of cells.

## Supplementary Materials

The following are available online, Figure S1: Effects of Pgp ligands on mitoxantrone permeability across BBB, Figure S2: Effects of MRP1 and BCRP inhibitors on doxorubicin transport across BBB, Figure S3: Effects of Pgp ligands on doxorubicin transport on Pgp-MDCK, MRP1-MDCK and BCRP-MDCK cells, Figure S4: Effects of Pgp ligands on dextran, sucrose, inulin and lucifer yellow permeability across BBB, Table S1: Phenotypic characterization of cells from patient number 1, 2, 3 by immunofluorescence analysis.
