# Supplementary material for: New Tetrahydroisoquinoline Derivatives Overcome Pgp Activity in Brain-Blood Barrier and Glioblastoma Multiforme in Vitro
Source: Molecules. 2018 Jun 9;23(6):1401. doi: 10.3390/molecules23061401 (PMC6099747; doi:10.3390/molecules23061401)
Supplement: Supplementary file 1 [file molecules-23-01401-s001.pdf]

Supplementary Materials

Supplementary Figure S1

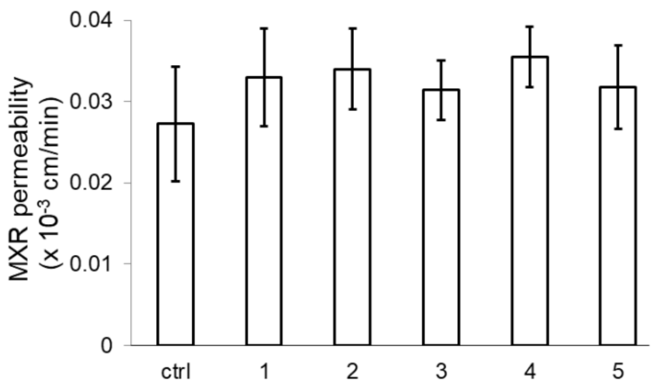

Supplementary Figure S1. Effects of Pgp ligands on mitoxantrone permeability across BBB

hCMEC/D3 cells were grown in the upper insert of Transwell devices for 7 days, then medium was replaced with fresh medium (ctrl) or with medium containing 1 nM of compounds **1-6** for 24 h. 10 μM mitoxantrone (MXR) was added in the last 3 h. The amount of mitoxantrone in the medium of the lower chamber was measured spectrofluorimetrically, in duplicates. Data are presented as means ± SD (n = 3).

Supplementary Figure S2

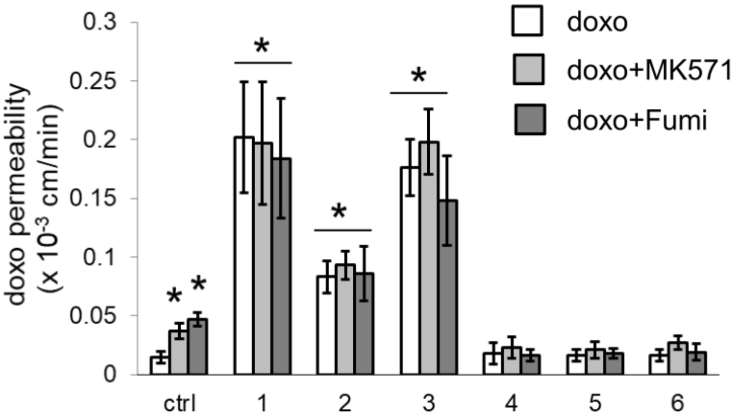

Supplementary Figure S2. Effects of MRP1 and BCRP inhibitors on doxorubicin transport across BBB

hCMEC/D3 cells were grown in the upper insert of Transwell devices for 7 days, then medium was replaced with fresh medium (ctrl) or with medium containing 1 nM of compounds **1-6** for 24 h. 5 μM doxorubicin (doxo) was added during the last 3 h, in the presence of 25 μM MK571, an inhibitor of MRP1, or 5 μM fumitremorgin C (Fumi), an inhibitor of BCRP. The amount of doxorubicin in the medium of the lower chamber was measured spectrofluorimetrically, in duplicates. Data are presented as means <u>+</u> SD (n = 3). Versus doxo: * $p < 0.01$.

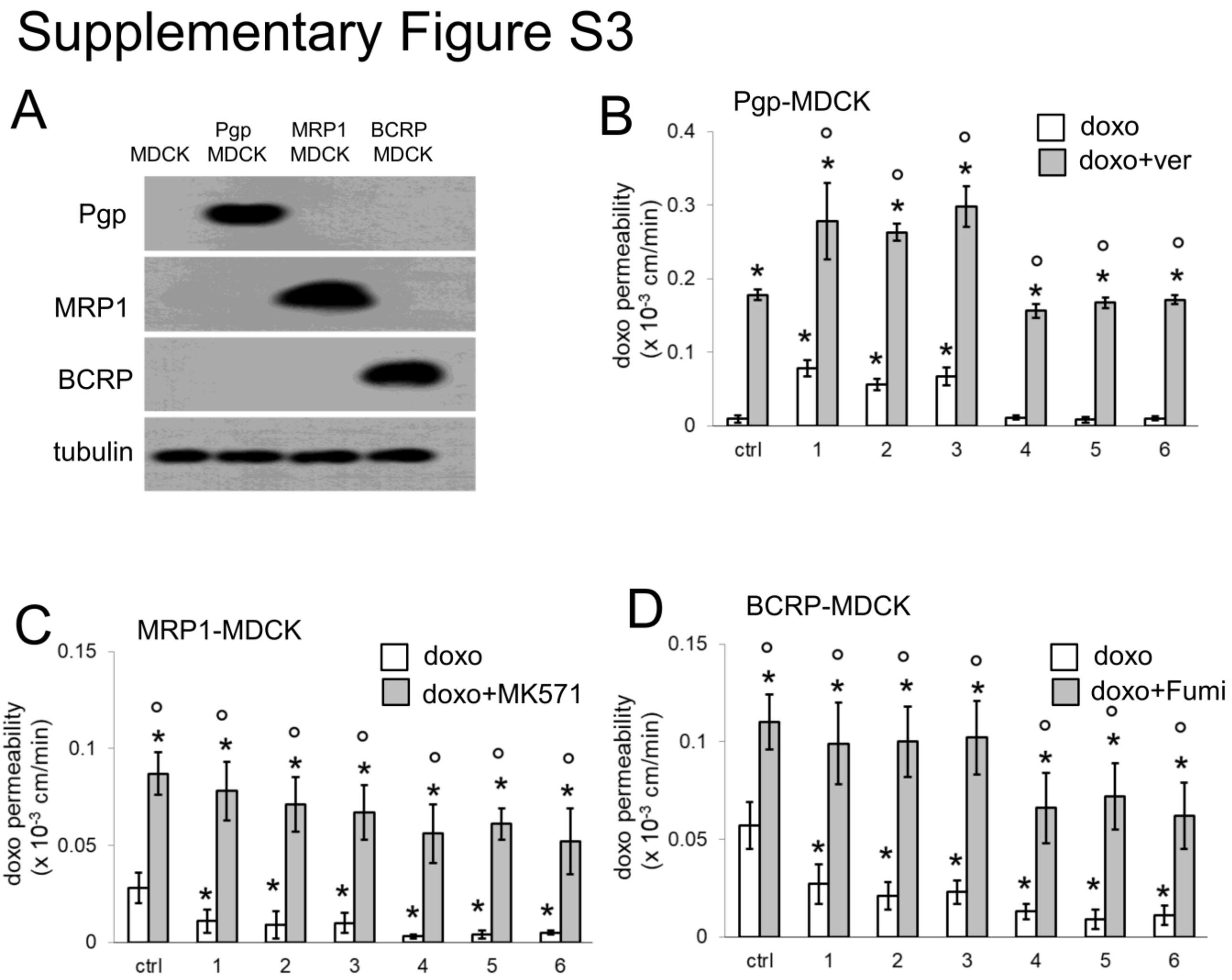

Supplementary Figure S3. Effects of Pgp ligands on doxorubicin transport in Pgp-MDCK, MRP1-MDCK and BCRP-MDCK cells

A. MDCK, Pgp-MDCK, MRP1-MDCK, BCRP-MDCK cells were lysed and immunoblotted with the indicated antibodies. β-tubulin level was used as control of equal protein loading. The figure is representative of one out of three experiments with similar results. **B-D.** Pgp-MDCK (panel **B**), MRP1-MDCK (panel **C**), BCRP-MDCK (panel **D**) cells were grown in the upper insert of Transwell devices for 7 days, then medium was replaced with fresh medium (ctrl) or with medium containing 1 nM of compounds **1-6** for 24 h. 5 μM doxorubicin (doxo) was added during the last 3 h. When indicated, 50 μM verapamil (ver), an inhibitor of Pgp, 25 μM MK571, an inhibitor of MRP1, or 5 μM fumitremorgin C (Fumi), an inhibitor of BCRP were added. The amount of

doxorubicin in the medium of the lower chamber was measured spectrofluorimetrically, in duplicates. Data are presented as means $\pm$ SD ($n = 3$). Versus doxo: * $p < 0.05$; doxo+ver/doxo+MK571/dox+Fumi vs doxo:° $p < 0.001$.

Supplementary Figure S4

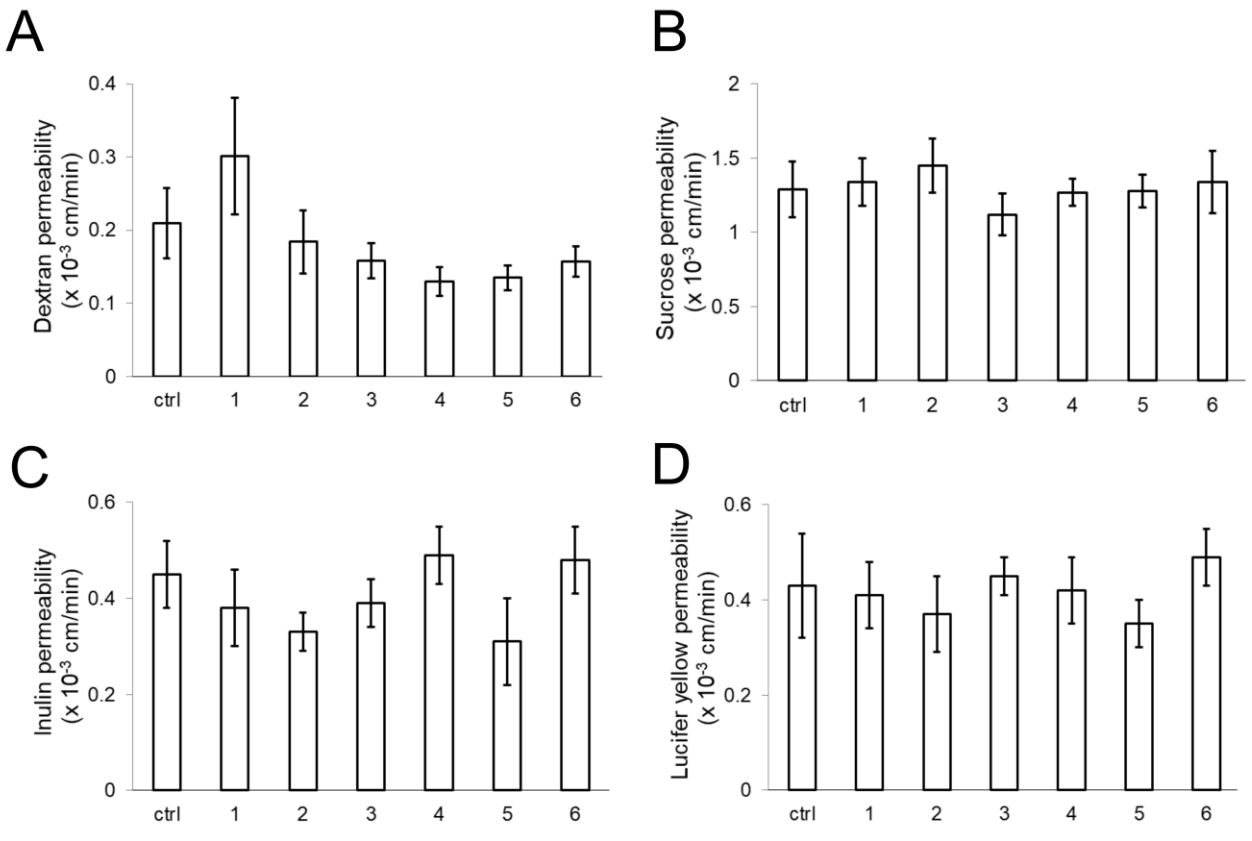

Supplementary Figure S4. Effects of Pgp ligands on dextran, sucrose, inulin and lucifer yellow permeability across BBB

hCMEC/D3 cells were grown in the upper insert of Transwell devices for 7 days, then medium was replaced with fresh medium (ctrl) or with medium containing 1 nM of compounds **1**-**6** for 24 h. 2 µM dextran-FITC (panel **A**), 2 µCi/ml [^{14}C]-sucrose (panel **B**), 2 µCi/ml [^{14}C]-inulin (panel **C**), 100 µM lucifer yellow (panel **D**) were added in the last 3 h. The amount of each compound in the medium of the lower chamber was measured spectrofluorimetrically (for dextran-FITC and luciferin yellow) or by liquid scintillation (for [^{14}C]-sucrose and [^{14}C]-inulin), in duplicates. Data are presented as means $\pm$ SD (n = 3).

Supplementary Table S1. Phenotypic characterization of cells from patient number 1, 2, 3 by immunofluorescence analysis

	NS	NS	NS	AC	AC	AC
**Marker**	**CV17**	**010627**	**Nov3**	**CV17**	**010627**	**Nov3**
**Nestin**	++	+	+	+	-	-
**CD133**	+	+	+	-	-	-
**Musashi**	+	+	+	-	-	-
**SOX2**	+	+	+	-	-	-
**EGFR**	+/-	+	+	+	-	-
**p53**	+	+	+	-	-	-
**GFAP**	-	-	-	+	+	+
**GalC**	+	-	-	+	+/-	++
